# Cultural Norms of Clinical Simulation in Undergraduate Nursing Education

**DOI:** 10.1177/2333393615571361

**Published:** 2015-02-17

**Authors:** Susan G. McNiesh

**Affiliations:** 1 San José State University, San José, California, USA

**Keywords:** decision making, education, professional, ethnography, knowledge construction, nursing education, performance, safety, patient, simulation, social participation, teaching / learning strategies

## Abstract

Simulated practice of clinical skills has occurred in skills laboratories for generations, and there is strong evidence to support high-fidelity clinical simulation as an effective tool for learning performance-based skills. What are less known are the processes within clinical simulation environments that facilitate the learning of socially bound and integrated components of nursing practice. Our purpose in this study was to ethnographically describe the situated learning within a simulation laboratory for baccalaureate nursing students within the western United States. We gathered and analyzed data from observations of simulation sessions as well as interviews with students and faculty to produce a rich contextualization of the relationships, beliefs, practices, environmental factors, and theoretical underpinnings encoded in cultural norms of the students’ situated practice within simulation. Our findings add to the evidence linking learning in simulation to the development of broad practice-based skills and clinical reasoning for undergraduate nursing students.

Simulation is a noteworthy addition to the clinical practicums of health care professionals in training, as it allows students to confront the real world of practice through a “near experience” in a safe, non-punitive environment. Moreover, scenarios can be constructed that simulate the unpredictability and complex character of the practice landscape. Thus far, clinical simulation has been embraced by the medical and nursing education community for a number of compelling reasons: increased student enrollment with more limited access to clinical sites (Schoening, Sittner, & Todd, 2006), increased patient acuity with higher use of technology with consequent complex care needs beyond the experiential reach of novice learners (Harder, 2012), and increased sophistication of computerized technology that makes the simulation environment mirror more closely the real conditions of the practice environment (Lasater, 2007).

## Literature Review

In a comprehensive and critical systematic review of simulation-based medical education (SBME) spanning four decades of research, McGaghie, Issenberg, Petrusa, and Scalese (2010) cited strong support for high-fidelity clinical simulation as an effective tool for learning performance-based skills. The researchers focused on listing best practices to maximize the educational benefit of simulation and also detailed the gaps in knowledge on simulation use. Although researchers applaud simulation as an effective learning strategy for the acquisition of skills in performing procedures, there is much less known about the efficacy of simulation use for social and relational skills such as communication, cultural sensitivity, and interdisciplinary collaboration, or to understand the processes that make up the learning environment of simulation (Dieckmann, Gaba, & Rall, 2007).

Using a grounded theory approach, Parker and Myrick (2012) aimed to analyze the social and psychological processes of student engagement in simulation activity, specifically to pedagogically inform nurse educators as to what might be deemed most appropriate in teaching nursing students in the 21st century. In their findings, the authors noted that simulating complex real-world scenarios could initially overwhelm students. The presence of the instructor bridged the gap in this situation and also decreased the threats to suspension of disbelief created by unrealistic aspects of mannequin use. In addition, they found that initially students were hampered by performance anxiety and fear of being judged, and that each of these factors could be modulated by faculty presence. In their discussion, the researchers proposed that educators use a two-step process to empower students: first providing students with a knowledge framework through the presence of the instructor within the simulation and subsequently offering fading support wherein the students take up a more independent role.

Harder, Ross, and Paul conducted a focused ethnography to articulate the culture of nursing simulation in an undergraduate nursing program. Using participant observation (*n* = 84), individual student interviews (*n* = 12), two faculty focus groups (*n* = 18), and individual faculty interviews (*n* = 2), the researchers have published two articles on their study: one focused on the faculty experience of engaging in simulation (Harder, Ross, & Paul, 2012), and the other focused on student perceptions of their learning within simulation (Harder, Ross, & Paul, 2013). The main theme in the article was instructor confidence within the simulation environment. Many faculty members were unfamiliar with simulation, yet were required to facilitate a simulation session with their clinical group during the semester. They felt their discomfort affected student involvement and learning. Recommendations included the need for an initial faculty development workshop as well as ongoing technical and pedagogical support for faculty enacting simulations.

The main findings of the second article (Harder et al., 2013) were students’ preference to be cast in active roles (e.g., the primary nurse) rather than the role of an observer, and to be cast in primary rather than supporting roles. In addition, the students preferred scripted guidance within roles and clear definition of role expectations. While these two articles noted some important themes, there has been a dearth of qualitative articles within the last 5 years that describe and integrate the roles, values, environment, and processes of simulation as a learning tool. Therefore, we chose to include Lasater’s study of clinical judgment within the high-fidelity simulation setting of one university’s nursing program published in 2007.

The aim of Lasater’s (2007) study was to examine the experiences of students during a university’s first semester of use of high-fidelity simulation, in particular describing the effect of simulation on the development of clinical judgment. Through observations (*n* = 39) and a focus group interview (*n* = 8) of junior-level nursing school students, the researcher condensed findings into five major codes: the strengths and limitations of simulation, paradoxical feelings, desire for more direct feedback from instructors, the value of connecting with others, and recommendations on how to better facilitate learning in simulation.

In the view of many educators and researchers, thus far simulation has been performed within a narrow vision of both tools and pedagogy (Harder, 2012; Schiavenato, 2009). Researchers note the need for more rigorous qualitative investigation into the processes within the simulation learning environment (Cook, Hatala, Brydges, Zendejas, & Szostek, 2011; Parker & Myrick, 2009) and more pedagogical scaffolding for simulation construction (Dieckmann et al., 2007; Schiavenato, 2009). In conclusion, it is precisely the *complexity* within the simulation learning environment and community of socio-relational practice that needs to be articulated to more fully realize the potential benefits of simulation technology.

## Methodology

An ethnography is a particularly appropriate mode of inquiry to gain understanding of participants’ actions and meanings as situated and participatory within a community of learning. When beginning their nursing education, students enter a new culture and as individuals are conditioned into this new social world. The culture guides their views and gives implicit structure to their experiences (Polit & Hungler, 1999). To some degree, the situated environment of simulation mirrors this culture, yet the conditions of time and place within the artificially constructed reality of a simulation laboratory have not been thoroughly articulated. This article reports on the findings of an ethnographic study of simulation within a nursing education program.

The overall goal of this qualitative research study was to ethnographically describe the culture of clinical simulation within the simulation laboratory of a baccalaureate nursing program in the western United States. We gathered and analyzed data from observations of simulation sessions as well as interviews with students and faculty to produce a rich contextualization of the relationships, beliefs, practices, environmental factors, artifacts of significance, and theoretical underpinnings within the simulation culture that served as barriers or facilitators toward learning safe, effective, team-based, and patient-centered care.

### Participants

Ninety-nine baccalaureate nursing students participated in the observational part of the study. They were a convenience sample of students engaged in their typically scheduled simulations across specialty topics, including medical/surgical, pediatric, and obstetrical content. A subset of students involved in the observed simulations volunteered to participate in small group interview sessions immediately following the simulations (*n* = 25). Our research team did not collect demographic data; however, during the time period of the study, the school of nursing was 83% women and 17% men. Eighty-five percent of the students were aged 20 to 29. Ethnicities included 32% White, 29% Asian (Asian Indian, Pakistani, Chinese, Vietnamese, Korean, and Japanese), 29% Pacific Islander (Filipino, Native Hawaiian), 8% Hispanic, and 1% Black (Program Director, personal communication, May 18, 2013).

### Research Team

At the time of the study I, as the primary investigator, had 3 years of experience facilitating simulation sessions with undergraduate students. The anthropology graduate students had varying levels of medical experience as one was an emergency medical technician, another had worked as a secretary in a hospital, and the third had no medical experience whatsoever. The nursing graduate student had participated in four semesters of simulation experience during her undergraduate program, and was currently employed as a school nurse. These students provided unique viewpoints from within and outside the field of nursing, and added depth to our data analysis as we were able to examine the culture from two insider perspectives (faculty and former student of simulation) and three outsider perspectives (anthropology students).

### Setting

The setting for this study was the simulation laboratory of a public university in the western United States. At the time of the study, the simulation laboratory consisted of one elongated room, with a retractable partition wall that separated the space into two rooms. Each room was a close facsimile to a hospital room, with the exception that there were four microphones hanging from the ceiling, as well as less obtrusive cameras positioned near the ceiling. There were high-fidelity mannequins occupying the hospital beds, infant sized, child sized, or adult, depending on the scenario focus for that session. Medical carts, monitors, and other equipment were organized around each bed area. There was an observation room sealed off from the rest of the lab by walls and two-way windows, allowing observers to see the scenario performed by the students in real time, while the activity was also recorded for possible playback during a debriefing session that immediately followed each scenario. One to two members of the research team observed scenarios from this vantage point along with the clinical faculty, a faculty person in charge of facilitating the simulation session, and a technology staff person.

### Protocol for Simulation

Each of the simulation sessions followed a similar protocol, though the content and specialty focus varied. Faculty would begin with an orientation in which the faculty would inform the students of the “ground rules,” primarily concerning confidentiality of content and behaviors during the scenario. This was followed by an orientation/familiarization with the physical setting of the laboratory, followed by a group brainstorming session wherein students were given a narrative report of the scenario and as a group needed to discuss how they would proceed to care for the patient. The simulation was then enacted, followed by a debriefing session.

The scenarios focused on care of an adult patient, a pediatric patient, or an obstetrical patient or newborn. Each session included three unfolding scenarios. Students were assigned roles as the primary nurse, an orienting nurse, the charge nurse, or family members accompanying the patient just prior to the enactment of each scenario.

### Data Collection: Observations

This research team performed observations of eleven 4-hour simulation sessions (including debriefing), typically with 1 to 2 observers at a time. Each observed session included a clinical group of 8 to 10 students (*n* = 99). We gathered data in the form of field notes and reflexive memos immediately following observations. The research team developed a set of guiding questions based on the literature, program objectives of the simulation laboratory, and student learning objectives for the undergraduate nursing program as a whole, to guide our observations (see Table 1). In particular, the team reviewed the Institute of Medicine’s report on quality and safety in health care (Cronenwett et al., 2007), and the results of the Carnegie Foundation for the Advancement of Educations’ national study of nursing education in the United States (Benner, Sutphen, Leonard, & Day, 2010). This use of a guiding framework for observation supports the notation by Hammersley and Atkinson (2005) that the ethnographer “will want to ask *what* to write down, *how* to write it down, and *when* to write it down” (p. 176) as a method to organize observations while they are in process.

**Table 1.** Protocol for Observations.

1. Be sure to articulate the physical elements (built environment), processes (social and procedural), relationships (RN to Patient and RN to RN, student to student, student to instructor— consider “separate but together” the debriefing session and the actual simulation), values (body language and demeanor), theoretical underpinnings (germ theory, bio-medicine, etc.), and artifacts/objects of significance (meaning) within the simulation culture.
2. Clearly describe contextual features that might act as barriers (consider things like role ambiguity or confusion, hierarchical relationships, educational difference, gender issues, cultural issues) or facilitators to students’ understanding of patient-centered care, teamwork and communication, and quality and safety in patient care. To “unpack” teamwork a bit: consider evidence of any of these: responsibility, accountability, coordination, cooperation, risk-taking, assertiveness, autonomy, mutual trust and respect—obviously we would be describing behaviors that we think “speak” to these values/traits.
3. Identify evidence of integration of cognitive knowledge (bringing their “book-learning” into their action-set), practical skills, and professional-ethical (do you see evidence of an ethical stance?) accordance within simulation and debriefing sessions.
4. Perhaps (especially at the beginning) 10 things that stood out to you.

### Data Collection: Interviews

We asked students to participate in small group semi-structured interviews directly following five of the simulation sessions (*n* = 25). One of the graduate assistants or the primary researcher conducted interviews lasting 45 to 60 minutes. We began by asking permission to audiotape the interview and then encouraged the participants to answer questions as fully as possible, emphasizing that there were no “wrong” responses to questions (see Table 2). In addition, each of the graduate assistants conducted a one to one semi-structured interview with a faculty member who had served as a facilitator of one of the observed sessions (*n* = 4).

**Table 2.** Interview Guide.

Note to interviewers: This is a semi-structured interview using an interview guide. Therefore, if the participants go on a relevant tangent, feel free to skip some questions and continue with the participant’s line of thought.
1. Describe the simulation experience from your point of view.
2. What does it feel like when you first enter the lab?
3. What do you consider is particularly valuable about simulation?
4. Tell me about your debriefing experience. (other question: What do you think is the purpose of the debriefing session?)
5. Can you tell me about a time that you learned something in simulation that you have since applied in the clinical environment?
6. Is it tough to get into the role? What helps? What hinders?
7. What would enhance your simulation experience?
8. Does simulation add something that isn’t present in your clinicals—and if so, what?
9. Can you think of an example of how simulation helps you learn about communication?
10. Can you think of an example of how simulation helps you learn the importance of working as a team?
11. What do you think of the statement, “It’s okay to make mistakes?”

### Protection of Human Subjects

Following the university’s institutional review board approval and with the permission of the clinical simulation instructor, one of the graduate students met with the students as a group at the beginning of their scheduled simulation session and briefly presented the purpose of the research, procedures, risks and benefits of participation, and asked students for written consent to participate. Students were informed if any members of the group did not wish to participate, that simulation would not be included in the study, although they would still enact the simulation as part of their regular coursework. The graduate student informed the students that the consent letter included a paragraph describing the opportunity to voluntarily participate in the small group interview following the simulation session. All students agreed to participate in the observations of simulation and between four and six students from each group self-selected to participate in the small group interview. Faculty members who volunteered to be interviewed were given a separate consent form to sign. Students and faculty that volunteered for the group interview received a US$10 coffee card.

A digital tape recorder was used to record interviews as wave files. We assured the students that observations would be anonymous and that we were mainly interested in collective processes rather than individual performance. We informed the students prior to their participation in interviews that their anonymity would be preserved within these sessions. Following professional transcription, I changed names of interviewees to ensure anonymity. Only myself, as the primary researcher, and the graduate assistants had access to data, and I destroyed all audiotapes upon completion of the study.

### Data Analysis

Data were stored and analyzed in constructed cloud-based documents, developed in a nested scheme that followed analytical outlines. This method was practical for two reasons: graduate research assistants did not have access to qualitative analysis software and we deemed it a more fluid structure for reconstructing themes as more data were analyzed. We started our data analysis during the initial observations; however, the data were collected over a defined period of 3 months. We began data interpretation with each researcher reading the texts of the field notes, reflexive memos, and interviews. Individual group members shared initial hunches in writing, followed by an in-person analytic session. We developed initial thematic categories at that time. Over the next few months, the group traded interpretive memos via shared document files, and began the process of developing cultural norms described in the thematic categories. These were drawn from described or observed actions during the simulations. The following semester, I continued with data analysis, using an iterative process of moving back and forth between identified parts (themes or norms) and whole, the whole examined in light of what I understood from the part and vice versa (Geertz, 1973/2000). I reviewed the data with two experienced qualitative researchers during that time.

### Rigor

The trustworthiness, or evaluation, of qualitative data can be assured through the criteria of credibility, dependability, confirmability, and transferability (Lincoln & Guba, 1985). In this project, credibility was assured through multiple means of triangulating data. First, we collected data through multiple forms—observations, interviews, and supporting records such as faculty provided learning objectives. Investigator triangulation occurred, as three anthropology graduate students, a graduate nursing student, and I made independent observations. Furthermore, we collected data over three semesters of students, assuring different levels of general experience in the nursing program as well as different amounts of time exposed to simulation. We assured dependability of the data through review by two external reviewers with background in qualitative methods. We developed a well-defined audit trail to assure confirmability, and enhanced transferability through a richly detailed account of findings.

## Findings

The culture that emerged was very particular to simulation. In fact, the environment more resembled an alternate world that students acted in, with an altered sense of time, self, and relationality. During the interpretive process, we developed a listing of cultural norms that described the processes, roles, relationships, and values that emerged from the data. The following list is not in particular order of importance, and should be read as much as possible with a sense of the functioning whole of experiential learning in simulation. Within these sections, student participants’ comments are identified by the student’s semester level. For example, sophomore-level nursing students are identified as either fourth- or fifth-semester students; junior-level students are identified as fifth- or sixth-semester students.

### Familiarizing to the Setting

The first cultural norm included the processes and beliefs surrounding familiarizing to the simulation setting. Like immigrants in a foreign country, the students determined there were ways of acting and communicating that were different than their ordinary “being a nursing student” but also considerably different than the habits and practices that they had learned during their clinical practicums. Students recognized that it took some time to assimilate to the setting and that they could more quickly inhabit and perform a role after a few semesters of exposure to the simulation experience. In this particular nursing program, the students attended the simulation laboratory twice in a semester.

In addition, as is necessary in clinical placements, the students needed to become familiar with the environment, the equipment, the routines, and the significance of representative signs. For example, as most simulations were dependent on the use of mannequins rather than human patients, the students needed to learn what was “normal” for the mannequin, how to differentiate normal mannequin behavior from human behavior. As students gained more experience with the simulation environment, they became more skilled performers:

I think it went really smoothly looking back to the other times we’ve done it, where it was just kind of a mess. And it was, you’re in there and, “Oh my God, I don’t know what to do.” And now it’s not so new to us, so we’re not as uncomfortable in the room, which I think takes a lot of the fear and anxiety away. (Fourth-semester nursing student)

Being uncomfortable in the room made the students’ actions more mechanical and less smooth. After a few sessions in the simulation laboratory, they developed routines of care, ways of interacting, and an understanding of the representative signs in the environment, that made their actions take on a smoother character.

### Enacting the Full Nursing Role

Students as experiential learners tended to take care of less complex patients within the real clinical setting, at least until their final semester. During interviews, faculty stated that in the hospital clinical setting, it was not safe for students to assume independent care of patients, and that instead students remained “tethered” to a nurse. A second observed cultural norm was that students were expected to act “as if” they were the fully licensed registered nurses within the scenario. This created many more possibilities for error but as one student expressed,

I think . . . we’re expected to have a little more independence in simulation because usually when we’re in the hospital, we’re following our nurses around and we just follow whatever they say. It’s not like we get to plan the care for the patient. We just follow what their plan is. But here we have to know; we have to formulate our own plan for the patient as compared to just following someone else’s. (Fifth-semester nursing student)

The student’s comments suggest that actions in the hospital alongside the nurse occurred without much clinical reasoning but rather as tasks assigned by the nurse. In contrast, the student developed the plan of care in the simulation laboratory.

Another student within the fifth semester added, “That’s the biggest thing is exercising your own critical thinking and, having the responsibility be yours rather than just following someone else’s lead.” Having to make decisions and then take responsibility for their actions were significant processes for the students, with a clear acknowledgment from all students during interviews that the debriefing session would be an opportunity for reflection on these processes.

### Making Mistakes in a Safe Place

Simulation provided students with an opportunity to become the responsible party in more complex clinical situations simulated to unfold as they might in the real-world setting. This segues to the third observed norm, that simulation was a safe place to make mistakes. This was encoded in the oft-stated remark by faculty to students that it was okay to make mistakes in the simulation scenario. We noted during our observations that this was part of the script in the orientation process that faculty enacted before each simulation.

The culture of simulation contained a modulated space between pretending and feeling the weight of situations that in the real world could include serious consequences. As one student noted, “There’s a lot of pretending going on so you kind of have this dichotomy of you’re pretending but you have to be serious at the same time.” The simulation environment allowed students to perform actions that if done incorrectly on real patients could cause irreparable harm. Learning from mistakes tends to stick, but in simulation there is a safe environment with “stand in” patients. Initially students might have felt badly about a mistake made in simulation. Over time and in reflection, the student felt the weight of a mistake less as described by this student:

During simulation, that’s when it feels the most real and that’s where all the pressure is and during simulation I don’t feel like it’s okay to make a mistake. But, it’s after simulation is over, once simulation has ended and debriefing happens and we walk home, that’s when I realize that is was okay to make a mistake ‘cause it was a learning experience. (Fifth-semester nursing student)

The alternate reality of simulation allowed for reflective time during the debriefing session wherein the individual had a deep focus on self and performance, as well as time during the simulation when the individual felt the “as if” pressure similar to a real situation. The student was disturbed about making a mistake during the simulation, but in the self-reflecting mode of debriefing, it felt permissible. The walk home provided the student with further physical and psychological distance so that the student could now frame the experience as learning.

When asked to further explain the dichotomy between learning in the simulation laboratory and learning in the clinical setting, another student stated,

I think knowing the environment had pretty much all of the tools and the choices that we needed and was very similar to what’s available in clinical. But, as far as the mannequin, I think that’s what kind of lowered the pressure for me a little bit knowing that it was just a mannequin. But, it still didn’t really change how I wanted to work and what I wanted to do as a student nurse in that scenario. You still need to get stuff done and it doesn’t really change the way you think and act. It’s just less pressure knowing that it’s just a mannequin. You’re not so hard on yourself if you were to make a mistake, because you’re not hurting a real person. (Fifth-semester nursing student)

This student identified the connection with a mannequin as different from the connection a student would make with a human, but recognized the mannequin “stand in” as a tool for learning in a safe space. The mannequin allowed the student to use prior understanding of patient care and the clinical environment to gain a viable solution to a clinical problem in the simulated setting.

One fifth-semester student discussed the possibility that faculty’s knowledge of common mistakes was used to structure the simulations. According to this student, it would be unsafe to use trial and error to structure students’ actions within the clinical environment. Rather, the student stated they would rely heavily on the nurse they were assigned to work with on the clinical unit. In deep contrast to their learning within the hospital clinical environment, the cultural norm within the simulation was that it was okay to make a mistake, and then the examination of the event would be opened up during the debriefing session that followed.

### Being Watched and Evaluated

A fourth cultural norm was the understanding that their performance in simulation was always under scrutiny, a greater awareness of “being watched and evaluated.” Interestingly, this sense of scrutiny went away in the heat of the simulation itself:

Walking into that environment . . . it was like a realistic environment. Things that we would find in clinical are there and timing of the reactions of the patient, like, the raise in the blood pressure and decreasing the O2 sat and the pain, it’s realistic even though the mannequin, it’s just a mannequin. That’s probably the most unrealistic thing there. And I still feel a lot of pressure just walking in. Because knowing I’m being watched by my instructor and everyone in the back. I didn’t think I would feel as much this time around, but I still felt the same amount of pressure because there’s actually more people in the back watching.

Interviewer: And you were remembering that in the middle of this scenario?

Student: Yeah, well, not in the middle of the scenario. In the middle of the scenario I was trying to get my assessment done for my situation. Yeah, when you’re caught up in the heat of the moment, it actually kind of does, I guess, it seems real. You try to get what needs to be done, done. But, when I walk into that environment I just feel like I’m taking care of a mannequin. (Fourth-semester nursing student)

There was pressure to perform, as students were aware that performances in simulation, though mistakes were allowed, were part of their clinical coursework.

In addition, they felt performance anxiety knowing that all would be “up for discussion” within the debriefing session. Although students and faculty agreed that the post-simulation debriefing sessions offer the greatest potential for student learning, there were some differences of opinion regarding whether nursing students should be judged based on their performance during simulation. Within this particular program, students were not graded based on their participation in nursing simulation; it was simply a required component of their clinical training. But does the absence of a grade mean that one’s peers and instructors were not evaluating your abilities? Even though one faculty member strongly asserted, “there’s no judgment,” her colleague disagreed, saying “no matter how we slice it or say it that, ‘oh, this is just feedback. Debriefing is not criticism; it’s not judgment.”’—“You can put lipstick on a pig, it’s still a pig. It’s judgment . . . Because it’s public.” Although both faculty members and students asserted that simulation was a safe environment in which it was okay to make mistakes, there was a public component that pressured students to perform to the best of their ability.

### Going Into a Mode of Skilled Performance

Another cultural norm was “getting past the plastic.” During the simulation itself, students often got swept away in the mood of the scenario (as noted in the previous section). They became less conscious of self, going into a mode of skilled performance that would then serve to increase their confidence level performing in situations that they might not have experienced within the clinical environment. One student describes this transition in consciousness:

In the beginning, I knew that there was going to be a mannequin inside. I knew that the professors were watching and that it was all just kind of a fake scenario. So, I didn’t have a whole bunch of pressure. I was just kind of more nervous that I was going to disappoint my clinical instructors since there were so many people watching with the whole environment. It was really realistic to a hospital setting. However, once the scenario actually started and once the mannequin started talking, I think it was that human voice behind the mannequin that actually made it so real that I almost forgot that the patient was just a plastic mannequin and I actually considered it, like, a real patient, like, that I’d be taking care of and what I would be doing and I got so caught up in the moment. So, I think it’s, like, in the beginning I knew it was just a mannequin and I was just more nervous. (Fourth-semester nursing student)

Another student continued the thought:

You learn about your abilities, it builds confidence when you’re in there, because you go in, and then you’re like “Oh my God, I’m not going to be able to do this.” And then, you kind of go into a mode, and you react, and you do things that you would not possibly be able to do, because we do so much observation, and we don’t do kind of hands on stuff.

In this student’s remarks, it appears that while slipping into a mode of skilled performance, the student was able to imagine performing in a similar way in the clinical environment wherein historically, as a student, was more of an observer.

### Valuing Reflection in Debrief

Culturally speaking, all of the students concurred that the debriefing cohered the simulation experience. Many students spoke of acting within the simulation, knowing that afterwards they would need to account for their actions during the debriefing, as noted in this exchange from two respondents in one group interview:

Respondent 1: I think it ties the whole simulation together. You kinda, otherwise you’re just kinda going in there and goofing around and if you come out you know, don’t say anything about it or just move on to the next one then you’re not really learning from it.

Respondent 2: You can make mistakes and think it’s correct and if you’re not debriefed then you’re just gonna go on and keep doing that.

Respondent 1: Yeah so it’s a really good way to break down you know, step by step and identifying maybe places where you could have made a different decision that would have affected the outcome in a more positive way so I think it’s essential. (Fifth-semester nursing students)

Debriefing was a focusing and organizing agent for these students’ learning. For the first student, the anticipation was that he or she would need to publicly declare decision-making rationale during the debriefing session, and that the debriefing created a space to break down clinical reasoning into reflective steps. The second student added an acknowledgment of the corrective feedback offered during debriefing.

Another student described the role as an observer of the simulation, without an active role within the scenario. In this case, the student was part of the group watching the simulation unfold from within the debriefing room via a live feed of the scenario:

I have to agree that when you’re watching the scenario, you’re in a relaxed environment, yet we’re still using our brains. We’re still critically thinking, you know, watching and thinking of ways of what they could do differently for the patient. And that’s pretty much it. It’s just still being engaged, but in a different way with less pressure. (Fourth-semester nursing student)

Even while watching from another room, the student was able to engage with the learning and consider what might be done differently if confronted with a similar situation. Faculty would often aid this process by providing students with a short list of focusing questions about what was happening within the simulation. We also observed faculty actively engaging observers during the debriefing session, asking for their reflections on the actions they observed the other students perform and what they might have done differently.

### Learning Collectively

Both within the simulation itself and within the debriefing sessions, it was a cultural norm for learning to be a collective and co-constructed activity. As noted earlier, students were expected to perform “as if” they were the real nurses during the simulation; however, unlike the real nurse in the hospital setting, in simulation the students worked in pairs, with perhaps one taking the role of a charge nurse or an orienting nurse alongside the student assigned to the role as the primary nurse. On a pragmatic level, this allowed for more students to participate directly in the simulation, but it also provided a serendipitous venue for group problem solving.

Students stated that because they were typically paired with a nurse in the hospital, they rarely had interaction with other students, and the nurse was clearly the lead decision maker in the clinical area. The difference when performing with their classmates was that it was a level playing field with each contributing to the developing clinical reasoning. Because every group of approximately 10 students that experienced simulation together were in the same hospital clinical group, the students had ample opportunity to develop relationships and respect for each other’s knowledge and abilities through the program. This was reflected in their comments about the debrief sessions; students relied on the feedback from other students within the debriefing session as valuable input on their performance:

And it [the debriefing] allows input from other people cause we don’t get that at clinicals cause not everyone’s watching you but here they’re able to see your manners of doing things and stuff so we can get input from other people as well as your professors and yourself too. (Fourth-semester nursing student)

During our observations, we were struck by the lack of competition between individuals in the clinical groups; there was a clear understanding that the simulation lab was a place for safe experimentation with an opportunity for constructive feedback from other students with a similar experiential level (as well as feedback from faculty). Safety was not only simulation as a safe place to make mistakes and not harm patients; safety also represented a learning environment where mistakes could be made, but students would not suffer academic repercussions for errors.

In addition, students in each of the three semesters of study described their collaborative practices within the simulation setting. This was likely enhanced by the fact that the students attended simulation sessions within their clinical group.

## Discussion and Implications

Lave and Wenger’s (2006) theory of situated learning places learning in both a historical and cultural context. The locus of learning shifts from a place of isolation inside of individual brains as the individual absorbs content to socially and contextually structured processes in a participatory framework that includes the idea of negotiated meanings. William F. Hanks noted in the foreword to Lave and Wenger’s book on situated learning, “Rather than asking what kinds of cognitive processes and conceptual structures are involved [in learning], they ask what kinds of social engagements provide the proper context for learning to take place” (p. 14).

Lave and Wenger (2006) use the term *legitimate peripheral participation* to describe the learning of a social practice, as the apprentice gradually takes up the master’s performance and makes it his or her own. Parker and Myrick’s (2012) empowering of students in simulation through fading support echoes the processes noted in legitimate peripheral participation, wherein the faculty acts as a guide and mentor earlier on in the simulation laboratory. In our study, we noted the group learning that often occurred during simulations, as students problem solved as a team during the action of the simulation scenario, or reflected on their own or a colleague’s performance during the debriefing session. However, in the background also appeared the clear guiding forces of the simulation faculty. Faculty supported learning in a number of ways such as clear maxims (“It is okay to make a mistake), expectations (“Perform as if you are the full fledged nurse”), and objectives and narratives that guided the simulation activity.

Numerous other authors have described simulation as a social practice (Dieckmann et al., 2007; Paige & Daley, 2009; Parker & Myrick, 2012). Dieckmann et al. defined the simulation experience as “a contextual event in space and time, conducted for one or more purposes, in which people interact in a goal-oriented fashion with each other, with technical artifacts (the simulator), and with the environment (including relevant devices)” (pp. 183–183). Faculty in charge of the simulation must carefully set up the boundaries leading into, out of, and through the simulation. Dieckmann et al. noted that rituals, such as a strict dress code or an overhead voice signaling when the simulation has ended, could play an important part in establishing this space. In our study, goal orientation and mutual purpose, identified through the stated or implied objectives of the simulated activity, allowed students to accept a wide variation in what passes as a believable scenario. Yet students acknowledged the shortcomings of the mannequins describing the lack of realism:

I noticed the automatic voice would sort of react based on how we moved the limbs or the body. So it was realistic in a sense. But then it took away any sense of humanness. It was just spitting out predetermined words. (Fourth-semester nursing student)

Benner et al. (2010) questioned the potential for developing skills of interpersonal communication via simulation scenarios. They cautioned that not only do mannequins lack the ability to display non-verbal expressions or subtly voiced psychological cues but also that real life holds more ambiguity than can be orchestrated in a simulation laboratory. However, Benner et al. did suggest that patient actors could fill the gap within the laboratory, a position echoed by our student participants.

Our study adds to the current body of literature on simulation as an effective pedagogy in clinical education by providing a deep description of the culture as articulated through interpreted cultural norms. Our findings describe how the environment was co-constructed, relying on the initial scaffolding by the faculty member but continually altered by the student participants, sometimes as individual constructions and sometimes by group consensus. Initially during the orientation phase, the faculty would often announce that the simulation laboratory was a safe place to make mistakes, and students were assigned to roles as nurses rather than student nurses. The students would take up these roles yet would transform them affectively over time, initially feeling the mistake as a point of shame but over time accepting the mistake as a lesson learned.

In considering the difference between the cultural norms of the hospital clinical environment and simulation, one important contrast our participants described and enacted was the concept of relationality and communication. Students constructed their clinical decision making alongside their colleagues during the scenario (as the primary nurse, orienting nurse, and charge nurse), and furthered this collective learning during the debriefing session that included other students who had observed the enacted scenario in real time from the debriefing room. This formation of a learning community was also evident in Lasater’s (2007) study on the development of clinical judgment in simulation. In that study, students emphasized the value of learning from the experiences of others as well as the value of learning collaboratively. They noted that the more public aspect of group learning broadened their perspectives and gave them more intervention options within the simulation and debriefing activities.

As is implicit in ethnographic work, a major limitation of this study was that it described the culture within a specific simulation setting. In addition, we could have gathered information over successive semesters rather than only observing students from three levels of undergraduate studies within the same semester. Moreover, ethnographic studies from other geographic areas could add to our understanding of simulation culture.

## Conclusion

Our study participants described an “as if” world, co-constructed for the sake of practicing the complex behaviors that are part of the professional nursing role. Within this world, students were free to take up the full role of the registered nurse and practice their clinical reasoning skills, undergirded by their prior knowledge and continually constructed rationales, to give patient care at a level of independence that would be unsafe for real patients in the clinical environment. Students recognized that the mannequins lacked many of the identifying features of a real patient and in spite of this were able to experience contextually the mannequins as tools for experiential learning. While enacting a scenario, students understood that they were under scrutiny by the other students as well as the faculty, yet they could also find themselves absorbed in the heightened action of the moment.

Debriefing remained central as a focusing event. Directly following the simulation, students often expressed disappointment in their performances, but during the space of the debriefing session, they were able to account for their actions and publicly reflect on their rationales and their performance. In a literature review specifically focused on debriefing, Neill and Wotton (2011) noted the importance of faculty guidance within the debriefing to develop a safe and trusting environment. These authors described the debriefing space as an examination of both process and outcome. Post-simulation debriefing provides a space and structure for students to openly reflect on their currently forming skills of practice. The findings of our study add a cultural context to the evidence linking learning in simulation to the development of effective clinical reasoning for undergraduate nursing students.
